# Development of the Neurochemical Architecture of the Central Complex

**DOI:** 10.3389/fnbeh.2016.00167

**Published:** 2016-08-31

**Authors:** George S. Boyan, Yu Liu

**Affiliations:** Developmental Neurobiology Group, Department of Biology II, Ludwig-Maximilians-UniversitätMunich, Germany

**Keywords:** insect, brain, central complex, development, neurochemicals

## Abstract

The central complex represents one of the most conspicuous neuroarchitectures to be found in the insect brain and regulates a wide repertoire of behaviors including locomotion, stridulation, spatial orientation and spatial memory. In this review article, we show that in the grasshopper, a model insect system, the intricate wiring of the fan-shaped body (FB) begins early in embryogenesis when axons from the first progeny of four protocerebral stem cells (called W, X, Y, Z, respectively) in each brain hemisphere establish a set of tracts to the primary commissural system. Decussation of subsets of commissural neurons at stereotypic locations across the brain midline then establishes a columnar neuroarchitecture in the FB which is completed during embryogenesis. Examination of the expression patterns of various neurochemicals in the central complex including neuropeptides, a neurotransmitter and the gas nitric oxide (NO), show that these appear progressively and in a substance-specific manner during embryogenesis. Each neuroactive substance is expressed by neurons located at stereotypic locations in a given central complex lineage, confirming that the stem cells are biochemically multipotent. The organization of axons expressing the various neurochemicals within the central complex is topologically related to the location, and hence birthdate, of the neurons within the lineages. The neurochemical expression patterns within the FB are layered, and so reflect the temporal topology present in the lineages. This principle relates the neuroanatomical to the neurochemical architecture of the central complex and so may provide insights into the development of adaptive behaviors.

## Introduction

Modular brain structures such as the insect mushroom bodies (MB) and central complex provide an ideal substrate for studies aiming to understand the developmental/genetic basis of neuronal function and behavior (Wegerhoff and Breidbach, 1992; Ito et al., 1997; Mizunami et al., 1997; Tettamanti et al., 1997; Meinertzhagen et al., 1998; Strausfeld, 1999; Farris and Sinakevitch, 2003; Ito and Awasaki, 2008). Located in the brain midline, the insect central complex comprises five major modules: the protocerebral bridge (PB), fan-shaped body (FB; or upper division of the central body), ellipsoid body (EB; or lower division of the central body), noduli (N) and lateral accessory lobes (LAL), and represents one of the most distinctive neuroarchitectures to be found among arthropods (Williams, 1975; Strauss et al., 1992; Strauss and Heisenberg, 1993; Renn et al., 1999; Heinze and Homberg, 2008; El Jundi et al., 2010; Young and Armstrong, 2010a,b; Boyan and Reichert, 2011; Ito et al., 2014; Pfeiffer and Homberg, 2014; Boyan et al., 2015; Koniszewski et al., 2016). The roles that these different modules play in organizing motor activity, in orientation, and in visual pattern memory and storage, have been demonstrated by both mutant analyses and lesioning studies (Huber, 1960; Ilius et al., 1994; Strauss, 2002; Liu et al., 2006; Neuser et al., 2008; Pan et al., 2009; Harley and Ritzmann, 2010).

In insects, the most conspicuous central complex module is the FB whose neuroarchitecture is characterized by layers of dendritic arbors accompanying a stereotypic columnar organization, both formed by the axonal projections of clusters of neurons located in the pars intercerebralis (PI) region of each protocerebral hemisphere (see Strausfeld, 2012). It has been proposed that the degree to which the insect central complex is elaborated in a given species is correlated with its lifestyle (Strausfeld, 2012; Koniszewski et al., 2016). Developmental studies have revealed that the intricate columnar wiring of the FB is established quite rapidly, but at stages which also vary with the lifestyle of the insect. In the grasshopper, it develops fully during embryogenesis (Boyan et al., 2008b, 2015), while in beetles, modules are added sequentially during larval development (Wegerhoff and Breidbach, 1992), and in flies, early-born neurons only become wired into the central complex during the pupal to adult transition (Renn et al., 1999; Young and Armstrong, 2010a,b; Riebli et al., 2013; Wolff et al., 2015). Despite this temporal diversity, the developmental mechanisms involved appear to be conserved and involve a process known as fascicle switching in which the axons of commissural neurons systematically decussate at stereotypic locations across the brain midline and in so doing generate the columnar neuroarchitecture required for adaptive behavior (see Boyan and Reichert, 2011; Boyan et al., 2015).

Paralleling this anatomical development is the development of the neurochemical architecture of the central complex. This is less well understood, but critical for understanding the role central complex circuits play in behavior. In the grasshopper, expression of neuroactive substances commences at a time when the neural stem cells (neuroblasts) are still present, allowing the participating neurons to be ontogenetically identified according to their lineage of origin (Boyan and Liu, 2014). This feature has led to central complex neuroblasts being shown to be multipotent in that they generate lineages in which a range of neuroactive substances are expressed (Boyan et al., 2010a). In *Drosophila*, by contrast, expression of such substances has only been documented in cell clusters of the adult brain (Kahsai and Winther, 2011) where no central complex neuroblasts remain (Ito and Awasaki, 2008), therefore making ontogenetic analyses more speculative.

In this review article, we use the grasshopper as a model system for central complex development. We focus on the FB and relate its neuroarchitecture to stereotypic patterns of axogenesis involving subsets of neurons from identified neuroblasts. We then show that neuroactive substances essential for the synaptic interactions within this system appear progressively during development according to a temporal topology that relates the position of neurons within a lineage to their age and axonal projection pattern. Temporal topology relates the structural to the biochemical neuroarchitecture of the FB and so may provide insights into the development of adaptive behavior.

## Discussion

### Neuroarchitecture, Neuronal Classes and Behavior

**Figure 1:**
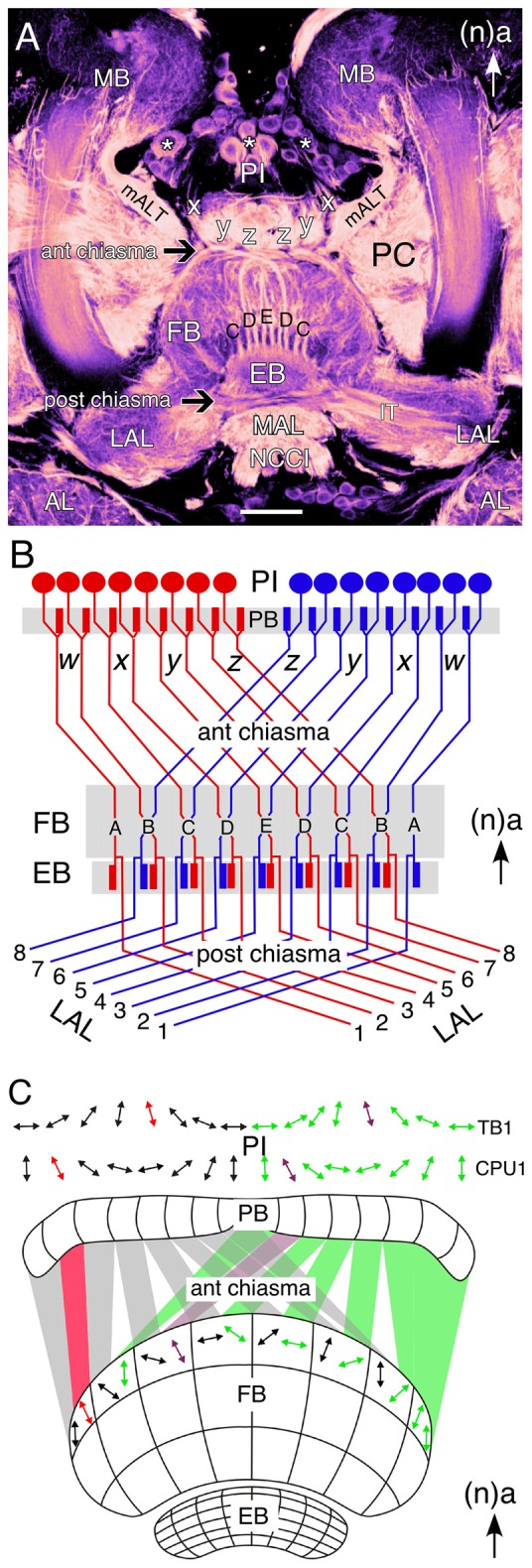
**Wiring of the central complex subserves information processing. (A)** Confocal image of a brain slice in an adult grasshopper (*Schistocerca gregaria*) following 8 B7 immunolabeling reveals the neuroarchitecture of the central complex between the bilateral mushroom bodies (MB) in the central brain. Neurons (white stars) of the pars intercerebralis (PI) direct axons via the *w, x, y, z* tracts into a chiasmal system (black arrow) anterior to the fan-shaped body (FB). These fibers then form columnar bundles (of which C, D, E, D, C are visible) in the FB and project further to the ellipsoid body (EB) and via a posterior chiasmal system (black arrow) laterally in the isthmus tract (IT) to the lateral accessory lobes (LAL). Note that the FB is also termed the upper division of the central body, and the EB the lower division of the central body (see Müller et al., 1997; Heinze and Homberg, 2008). White arrow points to anterior (a) according to the neuraxis (n) and is repeated for emphasis in all panels. **(B)** Wiring diagram (not to scale) illustrates the essential plan of axon projections from neurons of the PI via the protocerebral bridge (PB) into the *w, x, y, z* tract system of the left (red) and right (blue) protocerebral hemsipheres and hence via the anterior chiasmal system to form nine columns (A–E) in the FB and EB of the central brain. Axons subsequently exit the EB posteriorly and project via a posterior chiasmal system to the bilateral LALs. **(C)** Schematic (not to scale) illustrates the preferred polarization sensitivities (double arrows) of tangential (TB1) and columnar (CPU1) neurons in the PI of the grasshopper and the way these sensitivities are projected via the anterior chiasmal system to be represented within the columnar neuroarchitecture of the FB (CBU; see panel **B**). Other abbreviations: mALT, medial antennal lobe tract; AL, antennal lobe; MAL, median accessory lobe; NCCI, nervus corporis cardiaci I; PC, protocerebrum. Scale bar in **(A)** represents 100 μm. Panel **(A)** modified from Boyan et al. (2015) with permission; panel **(B)** modified from Williams (1975) with permission; panel **(C)** personal communication courtesy of U. Homberg.

The central complex comprises a set of five neuropilar modules—PB, FB (or upper division of the central body in the grasshopper), EB (or lower division of the central body in the grasshopper), noduli (N), LAL—of which the unpaired FB with its columns and stratified layers is the most prominent (Figures 1A,B; and see Williams, 1975; Heinze and Homberg, 2008; Strausfeld, 2012; Pfeiffer and Homberg, 2014). Four major neuronal types have been identified in the central complex of the grasshopper based on their projection patterns in and to its various modules (for details, see Heinze and Homberg, 2008; Pfeiffer and Homberg, 2014). Briefly, these are: (1) *Columnar neurons*, which connect single columns of the PB and/or the FB with the LAL or the noduli. Columnar axons project from the PB to the FB via the four fiber pathways known as the *w, x, y, z* tracts (Figure 1B; Williams, 1975; Strausfeld, 1976; Hanesch et al., 1989; Williams and Boyan, 2008). Within the FB, these axons form nine columnar bundles which will be considered in this review with respect to the developmental expression of various neurochemicals. (2) *Tangential neurons*, which innervate single layers of the FB, EB or PB and also interconnect the central complex with other brain regions (Strausfeld, 1976; Homberg, 1991; Müller et al., 1997). (3) *Pontine neurons*, which are intrinsic elements that connect specific columns and layers within the FB (Homberg, 1985; Hanesch et al., 1989). (4) *Amacrine neurons*, which are intrinsic to the FB. The one example recorded to date has a soma in the PI, a neurite in a *z* tract, and arborizations in a lateral hemisphere of the FB.

The insect central complex has been described as a multisensory neuropil processing visual, mechanosensory and olfactory signals on the one hand, while also serving as a premotor center, controlling walking, flight, acoustic communication and courtship on the other hand (see Strausfeld, 2012; Pfeiffer and Homberg, 2014). In *Drosophila*, specific subcompartments of the EB are involved in different aspects of spatial and landmark learning, orientation, and flight control (Ilius et al., 1994; Martin et al., 1999; Liu et al., 2006; Neuser et al., 2008; Pan et al., 2009; Triphan et al., 2010), while in the cockroach the FB regulates locomotory activity for negotiating barriers (Bender et al., 2010; Harley and Ritzmann, 2010). Of all the central complex modules in the grasshopper brain, the FB offers perhaps the clearest correlation between identified neuronal morphology, general neuroarchitecture, and information processing subserving behavior. This involves a particular form of visual information processing, namely sky polarization, which is fundamental to general navigation behavior in insects (see Wehner, 1989; Homberg et al., 2011; Weir and Dickinson, 2012). Electrophysiological recordings from identified tangential (TB1) and columnar (CPU1) neurons in the PI region reveal preferred polarization sensitivities representing a sky chart segmented into eight channels per brain hemisphere (Figure 1C). A comparison of preferred polarization sensitivities in the PI with those in an upper layer of the FB indicates a transformation en route which reflects the wiring plan for fibers entering the FB through the chiasmal system of *w, x, y, z* tracts (see Figure 1B, and see Williams, 1975).

### Development of the Central Complex

#### Organization of Neural Stem Cells

**Figure 2:**
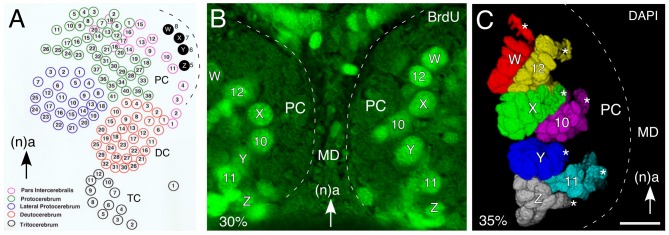
**Organization of neural stem cells (neuroblasts) in the early embryonic brain of the grasshopper. (A)** Schematic (not to scale) summarizes location of identified neuroblasts in the protocerebrum (PC), deutocerebrum (DC) and tritocerebrum (TC) of the left hemisphere of the grasshopper brain prior to mid-embryogenesis. Of these, W, X, Y, Z neuroblasts (shaded black) generate progeny contributing to the columnar organization of the FB of the central complex. Arrow points to anterior (a) according to the neuraxis (n) and is repeated for emphasis in all panels. **(B)** Fluorescent photomicrograph (false color) of a brain slice following bromodeoxyuridine (BrdU) incorporation at 30% of embryogenesis showing the bilaterally symmetrical distribution of identified mitotically active neuroblasts such as W, X, Y, Z in the median PC of each brain hemisphere. **(C)** 3D reconstruction based on *z*-stacks of confocal images following nuclear staining with DAPI at 35% of embryogenesis reveals the lineages associated with identified neuroblasts (white stars). Lineages appear in false colors. Other abbreviations: MD, median domain. Scale bar in **(C)** represents 35 μm in **(B,C)**. Panel **(A)** modified from Zacharias et al. (1993) with permission.

Topologically, the central complex belongs to the protocerebral neuromere of the brain (see Strausfeld, 2012). In the grasshopper, the brain is generated by approximately 100 bilaterally symmetrical pairs of neural stem cells called neuroblasts (Figure 2A), each of which is individually specified by molecular, positional and temporal cues (Zacharias et al., 1993; Reichert and Boyan, 1997; Urbach and Technau, 2003; Williams et al., 2005; Boyan and Reichert, 2011). Four of the neuroblasts (termed W, X, Y, Z) in each hemisphere play a key role in FB development in that their progeny establish the basic columnar organization of its neuropil. Genetic analysis reveals that a similar set of neuroblasts is found in each hemisphere of the developing brain of *Drosophila* (Izergina et al., 2009), and that these lineages contain the numerous columnar or small-field neurons that project to, innervate and interconnect the PB, FB, EB and noduli of the central complex (Ito and Awasaki, 2008; Izergina et al., 2009; Bayraktar et al., 2010; Young and Armstrong, 2010a,b; Riebli et al., 2013).

Since a neuroblast occupies a stereotypic location in the neuroepithelium (Figure 2B; Bate, 1976; Doe and Goodman, 1985a,b; Zacharias et al., 1993), and the lineages maintain their topological position in the CNS (Figure 2C), it is possible to profile such cell clusters temporally, biochemically and physiologically (Goodman et al., 1979, 1980).

#### Modular Organization of Lineages and Projections

**Figure 3:**
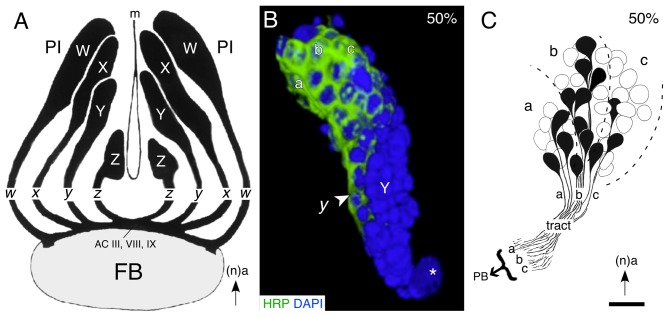
**Lineages of embryonic progeny and organization of projections into tracts of the central complex. (A)** Reconstruction based on serial brain sections shows the outlines of the W, X, Y, Z lineages of progeny and the associated tracts of axons projecting to identify commissural fascicles (ACIII, VIII and IX) of the embryonic (100%) FB. Arrow points to anterior (a) according to the neuraxis (n). **(B)** 3D reconstruction of a representative central complex lineage (Y) at 50% of embryogenesis based on *z*-stacks of confocal images following double labeling with the neuron-specific label HRP (green) and the nuclear stain DAPI (blue). Location of the Y neuroblast (white star), the initial *y* tract (white arrowhead), and subclusters (a, b, c) of mature (HRP-positive) neurons within the lineage, are indicated. Arrow indicates anterior (a) and applies to all panels. **(C)** Drawing from serial sections shows the soma locations and axon projections of individual cells from the a, b, c subcluster of cells at the tip of a representative central complex lineage (Z) at 50% of embryogenesis. Axon projections within the tract are topologically ordered according to the location of the somata within the lineage. Scale bar represents 15 μm in **(B)**, 25 μm in **(C)**. Panel **(A)** modified from Boyan and Williams (1997) with permission; panel **(B)** modified from Liu and Boyan (2013) with permission; panel **(C)** modified from Williams et al. (2005) with permission.

Examination of the cortical organization of the PI region of the brain reveals four discrete clusters of neurons (W, X, Y, Z) associated with the central complex (Figure 3A). This association is confirmed by the fact that axons from each of the clusters form a discrete tract (*w, x, y, z*) in which they remain, and then project to a small subset of commissural fascicles (AC III, VIII, IX) of the FB (Boyan et al., 1993; Boyan and Williams, 1997). The neuroblasts, their neuronal lineages, and the tracts they generate, can therefore be considered to represent individual modules or clonal units, consistent with the mechanism building association centers of the insect (Ito et al., 1997; Lee and Luo, 2001; Ito and Awasaki, 2008) and the vertebrate (Leise, 1990) brain. As in the ventral nerve cord, the progeny of a given lineage are generated according to a temporal order, and maintain their position according to birthdate within the cluster, so that the lineage acquires a temporal topology. Reconstruction of a central complex lineage after neuron-specific labeling confirms that early-born, mature, neurons at the tip of a lineage are already generating the initial tract while later-born, immature neurons nearer the neuroblast are yet to express the label (Figure 3B). Further, axon tracing reveals that there is a clear correlation between cell body location within such a lineage and the topology of axon projections into the associated tract en route to the primordial FB (Figure 3C).

#### Establishing a Neuroarchitecture

**Figure 4:**
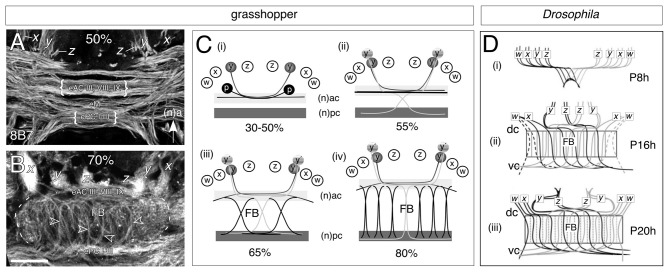
**Decussation of commissural axons from W, X, Y, Z lineages generates the columnar neuroarchitecture of the FB in the grasshopper (A,B) and *Drosophila* (C). (A)** Confocal image of a brain slice after 8B7 immunolabeling at 50% of embryogenesis shows axons from *x, y, z* tracts projecting into a commissural system of axons organized into parallel fascicles. Decussation of axons has not yet occurred but will involve fibers from embryonic anterior commissures (eAC) III, VIII, IX, the median commissure (eM), and embryonic posterior commissures (ePC) I-III (nomenclature from Boyan et al., 1993). Arrow indicates anterior (a) according to the neuraxis (n) and applies to panels **(A–C). (B)** Confocal image of a brain slice after 8B7 immunolabeling at 70% of embrogenesis shows axons from the *x, y, z* tracts entering eAC III, VIII and IX and then decussating to ePC I-III thus generating the initial fiber columns (open white arrowheads) of the FB. **(C)** Schematics (not to scale) illustrating the process of decussation to form the columns of the FB. The Y lineage here is representative for the general process. **(Ci)** Between 30% and 50% of embryogenesis, pioneer axons *(y)* from the Y lineage in each brain hemisphere fasciculate with the pioneers *(p)* of the anterior commissures but remain within their original fascicles as they cross the midline. **(Cii)** At approximately 55% of embryogenesis, later born neurons *(y’)* from the Y lineage follow the pioneers into the commissural system, but then decussate to enter the posterior commissural (pc) system. **(Ciii)** At 65% of embryogenesis, the process of decussation is repeated by axons from the other central complex lineages (W, X, Z). Decussation of bilaterally homologous axons occurs at stereotypic locations across the midbrain so that their crossing points mark the location of future columns. **(Civ)** At 80% of embryogenesis, the midbrain neuropil exapnds as dendritic arborizations increase (not shown) forcing the ac and pc commissural fascicles apart and making the columnar fiber bundles more orthogonal. The neuroarchitecture of the FB at these ages already resembles that of the adult brain (see Figure 1A). **(D)** Projection patterns (not to scale) of small-field neurons as summarized from anti-Echinoid labeling show that decussation in *Drosophila* during pupal development follows the same pattern as that in the embryonic grasshopper. Time is given in hours after puparium formation (Ph). Neurons originating from the right brain hemisphere (gray) are superimposed on the neurons from the left hemisphere (black). **(Di)** By P8 h double fiber bundles which we interpret as being equivalent to the *w, x, y, z* tracts of the grasshopper have projected to the midline and decussate to initiate the columnar organization of the primordial FB. **(Dii)** By P16h axons are decussating at specific locations across the midline thereby generating columns of the FB. **(Diii)** By P20 h, fibers project topographically, through the FB, to posterior neuropils (not shown). Commissures in *Drosophila* are named according to the body axis. Scale bar in **(B)** represents 35 μm in **(A,B)**. Panel **(A)** modified from Boyan et al. (2015) with permission; panel **(B)** modified from Boyan et al. (2008b) with permission; panel **(C)** modified from Young and Armstrong (2010b) with permission.

Developmental studies reveal that in both the grasshopper (Figures 4A–C) and *Drosophila* (Figure 4D), the intricate neuroarchitecture of the mature FB arises in a stepwise manner. Conserved cellular and molecular mechanisms, which may even extend to vertebrates (Arendt and Nübler-Jung, 1996; Tomer et al., 2010), establish an initial orthogonal axonal scaffold in the brain (Figures 4A,Ci,Di; see Reichert and Boyan, 1997). At these early stages, commissural axons remain tightly bundled within their fascicles. Significantly, the pioneers of the *w, x, y, z* tract system utilize the existing axonal scaffold previously founded by the commissural pioneers in order to navigate the brain midline (Williams and Boyan, 2008). As these *w, x, y, z* tract pioneers remain committed to their commissural fascicles, axonal reorganization must involve cells from each lineage which are either born later, or generate axons later.

The subsequent transformation of this initial orthogonal ground plan into the mature chiasmal/columnar neuroarchitecture involves a topographic decussation of axons (also known as “fascicle switching”) across the cerebral midline. Homologous clusters of later-born neurons from each protocerebral hemisphere redirect their axonal growth cones from an anterior to a posterior commissural subsystem (dorsal to ventral according to body axis) at stereotypic locations to generate the columnar neuropil of the mature FB (Figures 4B,Cii–iv,Dii,iii; Boyan et al., 2008b; Ito and Awasaki, 2008; Young and Armstrong, 2010a,b; Riebli et al., 2013). The points at which de- and re-fasciculation occur ultimately hinge the columnar system of fiber bundles within the FB. In both systems the columns subsequently thicken as progressively more axons decussate, and the gap between the commissural subsystems widens as the dendritic arbors from other innervating neurons expand. This leads to the staves assuming a progressively more orthogonal orientation (Boyan et al., 2008b, 2015). In the grasshopper, the neuroarchitecture of the chiasmal system at 70% of embryogenesis already resembles that of the adult (see Figure 1A).

Since the decussation follows topographically—axons from medial lineages (e.g., Z) project furthest across the midline while those from more lateral lineages (e.g., W) project the least—the data argue for there being a signal gradient, or a specific label, distributed along the medio-lateral axis that instructs neurons at specific locations (and therefore ages) within the lineage as to where to make their axons decussate (see Boyan et al., 2015). The pattern of axogenesis is also remarkably similar across species despite the fact that in *Drosophila* the columns are generated postembryonically from secondary neuron populations and not from primary embryonic populations as in the grasshopper, again arguing for a conserved molecular signal.

### Neuroactive Substances and Neuroarchitecture

#### Developmental Expression Patterns

**Figure 5:**
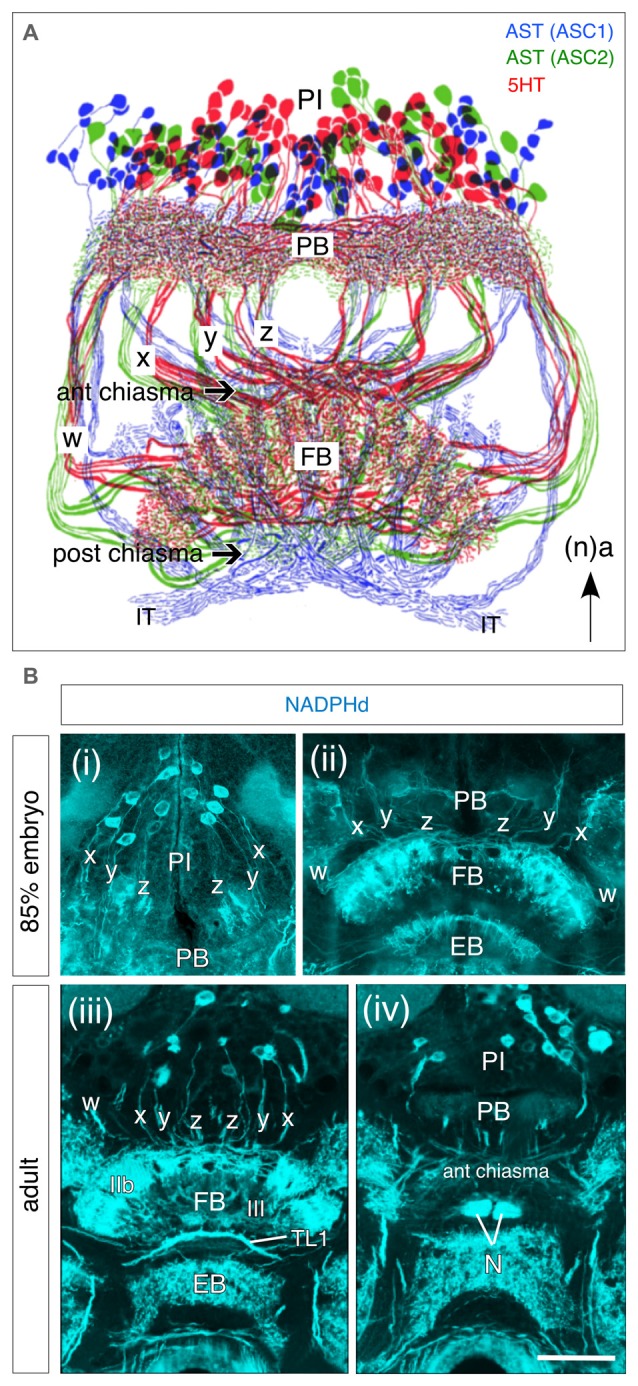
**Neurochemical architecture of the central complex based on projections from progeny of W, X, Y, Z lineages. (A)** Reconstruction reveals a highly conserved pattern of projections from bilateral populations of columnar cells in the PI to the FB via the *w, x, y, z* tracts following immunolabeling against serotonin (5HT, red), and allatostatin (AST; ASC1 subpopulation, blue; ASC2 subpopulation, green). Axons form columnar fiber bundles within the FB and then project via the IT to the LAL (not shown). Arrow indicates anterior (a) according to the neuraxis (n) and applies to all panels. **(B)** Frontal sections through the brain of an 85% embryonic **(Bi,ii)** and adult **(Biii,iv)** grasshopper following diaphorase (NADPHd) staining reveal nitric oxide (NO)-positive cell bodies from pontine and columnar neurons in the PI with projections into the *w, x, y, z* tracts and then to the FB via the PB. **(iii)** Note staining of columnar neurites in layer III of the adult FB. Axonal fibers of tangential TL1 neurons are visible between the FB and EB. **(iv)** Neurites from columnar neurons project from the PB into the anterior chiasma (according to neuraxis) and then to the noduli (N). Scale bar in **(Biv)** represents 1mm in **(A)**, 25 μm in **(Bi,ii)**, and 115 μm in **(Biii,iv)**. Panel **(A)** modified from Homberg (1991) and Vitzthum et al. (1996) both with permission; panels **(Bi,ii)** modified from Herbert et al. (2010) with permission; panels **(Biii,iv)** modified from Kurylas et al. (2005) with permission.

Association centers in the insect brain such as the central complex possess a conspicuous cellular neuroarchitecture: that of the FB for instance, that has been shown to comprise not only two systems of columnar tracts, but also multiple layers involving projections from pontine cells of the PI and tangential fibers from cells elsewhere in the protocerebrum (PC; Williams, 1975; Strausfeld, 1976, 2012; Müller et al., 1997; Heinze and Homberg, 2008; El Jundi et al., 2010). This neuroarchitecture is paralleled by a neurochemical architecture as revealed, for example, in the axonal projections of subsets of neurons from the PI expressing serotonin (5HT) and allatostatin (AST) in the adult (Figure 5A), or by diaphorase (NADPHd) staining as a marker for nitric oxide (NO) already at 85% of embryogenesis (Figures 5Bi,ii). This congruence of anatomical and neurochemical architecture can also be demonstrated using a range of other molecules (Homberg, 2002; Herbert et al., 2010; Pfeiffer and Homberg, 2014; Beetz et al., 2015) whose contributions to adult behaviors are steadily being revealed (Seidel and Bicker, 1996, 2002; Homberg, 2002; Nässel, 2002; Winther et al., 2006; Kahsai and Winther, 2011; Kunst et al., 2011). The question at the center of this study is when and how these stereotypic neurochemical projection patterns arise during embrygenesis. Clearly the central complex has adult characteristics well before hatching (see Boyan et al., 2015) and we need to look earlier for the origins of its neurochemical architecture.

Neuronal networks in the grasshopper have been shown to be activated by neurochemicals prior to the appearance of the adult behavior (Stevenson and Kutsch, 1986) and in the central complex, their expression begins during embryogenesis at a time when the mother neuroblasts are still present (Herbert et al., 2010; Boyan and Liu, 2014). This is a considerable advantage because if the original neuronal population can be age-profiled (see Figures 3B,C), expression can be traced to the temporal topology of a lineage so that a biochemical “fingerprint” of the central complex at different embryonic ages can be generated. This may, in turn, provide an insight into the developmental origins of central complex-related behaviors.

**Figure 6:**
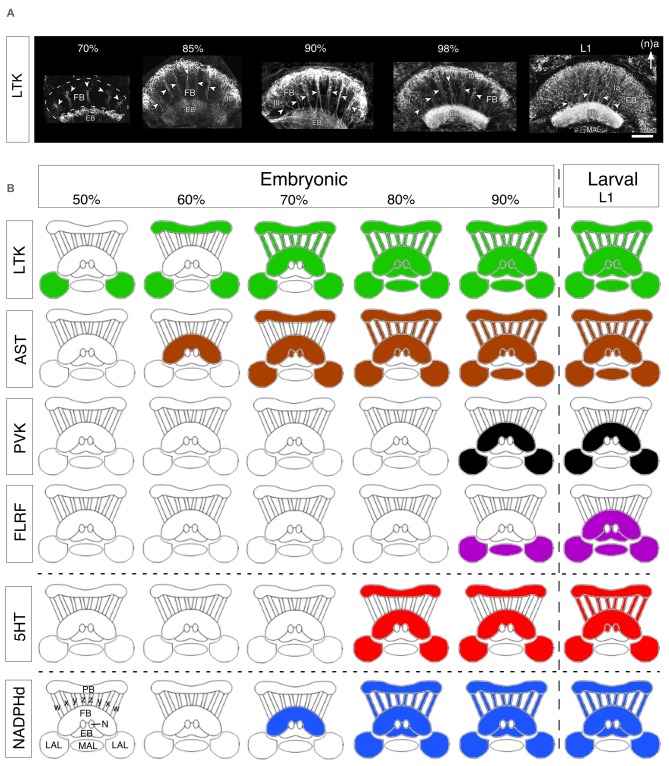
**Developmental expression of neurochemicals in the central complex. (A)** Confocal images of Locustatachykinin (LTK) immunolabeling in the FB and EB through successive embryonic (%) and postembryonic (larval stage 1, L1) stages. At 70% of embryogenesis, LTK labeling is present in the initial fiber columns (white arrowheads in all panels), region III of the FB, and an upper layer of the EB. At 85%, LTK labeling has expanded to include the Ib layer of the FB and most of the EB. At 90% of embryogenesis, the intensity of LTK labeling increases in layer Ib, fiber columns, and region III of the FB. The doublet structure of fiber columns (white arrowheads) is clearly revealed (see Figure 1B). Toward the end of embryogenesis (98%), LTK labeling in the EB increases to levels found postembryonically (L1). At L1, LTK labeling in neuropilar subregion Ia of the FB increases significantly reflecting the expanded dendritic arborizations there. Arrow indicates anterior (a) according to the neuraxis (n) and applies to all panels. Other abbreviations: MAL, median accessory lobe. **(B)** Schematics (not to scale) reveal stepwise expression of neurochemicals in the central complex over successive embryonic (%) up to the first postembryonic (L1) stages. Colors indicate the presence of immunoreactivity. Hatching from the egg is indicated by the vertical dashed line. Data are grouped into representative neuropeptides (LTK, AST, periviscerokinins and pyrokinins (PVK, PK), FLRFamide), a classical transmitter (5HT, 5HT), and the synthesizing enzyme (NADPHd) for the endogenous messenger NO. Modules of the CX are shown in outline and represent, as labeled at bottom left: PB, protocerebral bridge; *w, x, y, z* tracts; FB, fan-shaped body; N, noduli; EB, ellipsoid body; LAL, lateral accessory lobes; MAL, median accessory lobe. Scale bar in **(A)** represents 50 μm. Panels **(A,B)** modified from Herbert et al. (2010) with permission.

Here we consider a small range of neuroactive substances with respect to their development in the central complex of the grasshopper. These fall into three groups: (a) the neuropeptides, locustatachykinin (LTK), leucokinin-1 (LK-1), AST, periviscerokinin/pyrokinin (PVK/PK), FLRFamide (FLRF); (b) a classical transmitter, 5HT; and (c) a gas, NO. While by no means exhaustive, we suggest that the embryonic expression patterns of this subset of substances (Figure 6; and for greater detail, see Herbert et al., 2010) nevertheless reflect the major trends in neurochemical and neuroanatomical organization of the developing central complex.

##### Locustatachykinin (LTK)

Locustachykinins have a variety of effects in physiological and pathological events (as neurohormones and neuromodulators) which may vary substantially depending on the activation of different receptor subtypes (see Severini et al., 2002). A relatively early expression of tachykinins has been reported for a range of insect nervous systems (Nässel and Winther, 2002; Nässel, 2002) and there is some evidence that members of the tachykinin family can act as neurotrophic factors (Satake et al., 2003), perhaps comparable to their role in the development of vertebrate respiratory networks (Wong-Riley and Liu, 2005).

Vitzthum and Homberg (1998) distinguish six distinct types of LTK-immunoreactive neurons with ramifications in the central complex of the adult grasshopper: four columnar neuron groups (LTC I-IV), and two tangential neuron groups (LTT I-II). The LTC I cells are located anteriorly (according to the neuraxis) in the PI, project into the columnar tracts of the FB, and give rise to arborizations in the PB, EB and the lateral bulb (LBU). LTC II neurons are also located anterior to the PB and project via the columnar tracts to arborize in the PB and LBU. The columnar LTC III cell group is composed of eight large and 16 small neurons anterior to the PB with arborizations also in layers I and II of the FB. LTC IV neurons project into the posterior (ventral in Heinze and Homberg, 2008) groove and arborize in layers I and IIa of the FB. The tangential LTT I neurons located in the inferior median PC project to the LAL and ramify in the LBU. They then project through the isthmus tract (IT) to the EB. LTT II are posterior PI neurons which richly arborize in the PB, terminating in the posterior median PC of the contralateral brain.

In the developing central complex, LTK immunoreactivity is already detected at 50% of embryogenesis in tangential neurons of the LALs (Figure 6B). These neurons have their terminals in the area of the developing EB, posterior to the future FB. The pattern is consistent with these fibers belonging to the tangential LTT I neurons described by Vitzthum and Homberg (1998) in adults. At 60% of development, an additional tangential projection system belonging to the LTT II group begins to express LTK. The stained structures observed in the LALs colocalize to the adult LBU and the IT. At this stage there is also a relatively weak LTK immunostaining in the PB. LTK immunoreactivity in columnar neurons is first observed at 70% of embryogenesis (Figure 6A). Strong immunoreactivity is present in the PB, the *w, x, y, z* tracts, and the EB. LTK-positive arborizations are present in the anterior part of layer Ib of the FB, consistent with these being from the LTC I, II neurons of the adult. At 85% of embryogenesis, LTK immunoreactivity is seen in arborizations within the FB, and appears in the median accessory lobe (MAL), and in the posterior part of the noduli. This immunoreactivity subsequently becomes more intense (especially in the EB), but the overall pattern does not change. In early postembryonic stages, immunoreactive arborizations originating from the LTC III columnar cell group appear in layers I and II of the FB, and there is also expression in the posterior groove indicating LTK expression in LTC IV neurons. The immunoreactive pattern at this stage already resembles that of the adult (see Vitzthum and Homberg, 1998).

##### Allatostatin (AST)

The ASTs, termed schistostatins (Schoofs et al., 1998), have previously been identified in the adult central complex (Vitzthum et al., 1996) and are pleiotropic in function. As neurohormones they inhibit juvenile hormone (JH) synthesis by the corpora allata, while as inhibitory substances they are also involved in the modulation of muscle contraction, and the maturation of neural circuits (Rankin et al., 1998; Dircksen et al., 1999; Kreissl et al., 1999). JH has been shown to regulate aggregation behavior and olfactory processing in *Schistocerca gregaria* (Ignell et al., 2001). The expression of ASTs in the brain could therefore be consistent with a role in the maturation of synaptic pathways for locomotion, stridulation, and antennal-based behaviors known to involve the central complex (Bender et al., 2010; Harley and Ritzmann, 2010; Kunst et al., 2011).

AST expression in the central complex first appears in tangential projection neurons at the 60–65% stage (Figure 6B). Based on their morphology and location, these first AST-immunoreactive fibers are likely to be projections of AST one neurons (see Figure 5A). There is no AST immunoreactivity in the PB, *w, x, y, z* tracts or LALs at this stage. After 70%, further AST-immunoreactive tangential projections appear in the FB, with strong immunoreactivity in the PB, but still not in the columnar *w, x, y* and *z* tracts, suggesting that these PB projections belong to type IV tangential projection neurons with cell bodies located dorso-laterally of the PB (see Vitzthum et al., 1996). AST-immunoreactive horizontal fibers branching off in layers Ib and IIb are consistent with their being the AST three neurons described by Vitzthum et al. (1996). The AST two projection system is also labeled in the IT, and in layer IIB and the columnar system of the FB (see Figure 5A). From 80% onward all four AST tangential projection systems are immunoreactive, as are the columnar neurons and their processes in the PB, the *w, x, y* and *z* tracts, the noduli, and the LALs. Based on their labeling in layers Ia, Ib of the FB, it is likely that the ASC one neurons are the first to express AST in the columnar system. During further development, additional labeling appears in the MAL and in branches within the FB consistent with AST expression in the columnar ASC one neurons. The density of the AST-immunoreactive arborizations subsequently increases so that the pattern in first instars is essentially the same as in the adult.

##### Periviscerokinins and pyrokinins (PVK and PK)

PVKs and PKs are the major neuroactive components of the neurosecretory organs of the abdominal ganglia, and are also present in interneurons of the CNS (such as the columnar neurons in the cockroach central complex) in several insect species (Eckert et al., 1999) In the adult grasshopper, Herbert et al. (2010) report PVK/PK immunoreactivity in the tangential system, in the LAL and in all CB layers. Columns of the ascending tangential system of the FB also show immunoreactivity. Two immunoreactive cell groups located in the inferior PC project axons through the LALs into the FB. The first group projects via the ipsilateral LALs into layer Ib of the FB, the second group projects through the ipsilateral LAL into the contralateral LAL and into the FB.

PVKs appear relatively late in embryogenesis (90%) in the cell group projecting via the LAL into layer Ib of the FB (Figure 6B). However, strong PVK/PK staining is seen earlier (80%) in the lateral PC and in neurosecretory pathways. Staining is absent in the columnar PI neurons, in PB fibers, in the EB and in the noduli. The immunoreactive pattern in early postembryonic stages already resembles that of the adult (Clynen et al., 2009; Herbert et al., 2010). While effects on neurogenesis or neuronal differentiation are not known, PVKs are known to have myomodulatory and diuretic effects (Predel and Wegener, 2006), so that their postembryonic appearance could conceivably be associated with olfactory inputs regulating the transition from a yolk to a vegetative diet after hatching.

##### FLRFamide

FLRFamide-like peptides are expressed primarily in tangential projection neurons of the adult central complex. These neurons project into the accessory lobes and then via the IT into the EB and layer I of the FB (Homberg et al., 1999). FLRFamide-like immunoreactivity in the central complex appears only relatively late (90%) during embryogenesis and then in fibers of the LALs and MAL (Figure 6B). Other cerebral regions, however, show intensive FLRFamide-like immunoreactivity already at 65% of embryogenesis. In the first instar, weak staining is present in tangential fibers which follow columnar tracts of the FB and EB and becomes stronger during subsequent instars. As a neurohormone, FLRFamide is released by the corpora cardiaca-corpora allata system and regulates the heartbeat, influences the contraction of leg muscles (Robb and Evans, 1990) and has myoinhibitory effect on the locust oviduct (Peeff et al., 1994). These functions are more likely to be associated with aggregation and approaching sexual maturity in free-living (postembryonic) developmental stages, but a specific role with respect to the central complex is yet to be determined.

##### Serotonin (5HT)

5HT immunoreactivity has been extensively described in the central complex of adult *Schistocerca* (see Homberg, 1991, 2002; Herbert et al., 2010). Several serotonergic small-field neurons (S1 cells) of the PI project via the PB and the columnar *w*, *x*, *y* and *z* tracts into layer III of the FB, and to the noduli (see Figure 5A). Five other large-field neuron pairs/groups constitute the tangential serotonergic projection system of the central complex: the S2 cells project to the PB and the posterior optic tubercules; the S3 neurons of the inferior PC run through the LAL and the IT into layer Ia of the FB; the S4 neuron pair in the fronto-median PC gives rise to varicose branches in layer Ib of the FB; the S5 neuron pair in the posterior PI innervates layer Ia of the FB and the S6 pair projects into the contralateral LALs where their terminals arborize extensively.

In the grasshopper, appreciable 5HT immunoreactivity appears in the central complex only after 75% of embryogenesis, but then intensifies significantly during further development (Figure 6B). 5HT appears first (75–80%) in tangential projections in a manner similar to that of members of LTK and AST peptide families, and can be first detected in the columnar system shortly before hatching. At the 80% stage, tangential projection neurons (the future S2-S6 group) begin to express 5HT in their projections to the PB (S2), and via the LALs into the FB (S5). Serotonergic fibers are also present throughout the FB indicating that 5HT is present in the S3, S4 and S6 groups. All these serotonergic tangential projection neurons are immunoreactive simultaneously. By contrast, 5HT immunoreactivity is yet to be detected in the columnar *w, x, y* and *z* tracts and in PI neurons. In the late embryo (90–95%), the staining intensity rises in the tangential projection neurons as the neuropilar volume of the central complex expands due to increasing fiber density from additional ingrowing neurons. 5HT immunoreactivity in the columnar system can be first detected at 99%, shortly before hatching, and the overall pattern is then very similar to that of the L1 and adult stages, although the full complement of S1 immunoreactive cells (~60 cells after Homberg, 1991) is not evident yet.

In adult insects, 5HT which is involved in regulating circadian rhythms (Saifulla and Tomioka, 2002; Yuan et al., 2005), plays a role in odor-dependent behaviors (Kloppenburg et al., 1999), is necessary for spatial learning and memory (Sitaraman et al., 2008; Zars, 2009), modulates aggression (Edwards and Kravitz, 1997; Diereck and Greenspan, 2007; Johnson et al., 2009) and in the grasshopper, raised serotonergic levels which mediate the phase change from solitary to gregarious (Rogers et al., 2004)—all behaviors in which the brain, and with it the central complex, is likely to be involved. Neuroactive substances are known to be hierarchically organized (Gammie and Truman, 1997) and developmentally, 5HT has been considered to function as a general coordinator of neurogenesis, axogenesis and cellular and biochemical differentiation (Turlejski, 1996; Gaspar et al., 2003; Richards et al., 2003; Vitalis et al., 2007; Filla et al., 2009). Serotonergic neurons have been shown to require chemical signals in order to become functionally active (Condron, 1999) including 5HT itself (Sykes and Condron, 2005) suggesting that feedback as well as feedforward networks are active in the developing grasshopper nervous system.

##### Nitric oxide (NADPHd)

NADPHd activity is present in the adult MB and central complex (O’Shea et al., 1998; Kurylas et al., 2005; Siegl et al., 2009) and NO has been linked to cell proliferation, retinal patterning, axogenesis, synaptogenesis and neuronal maturation in developing insect nervous systems (Kuzin et al., 1996; Truman et al., 1996a,b; Ball and Truman, 1998; Gibbs and Truman, 1998; Seidel and Bicker, 2000). A detailed analysis of NO (NADPHd) immunolabeling associated with the central complex of the adult grasshopper is presented in the study by Kurylas et al. (2005). Six neuron types (about 170 neurons) involving tangential, pontine and columnar projection systems were identified: (a) *columnar neurons*. About 50 neurons from the posterior PI (according to the body axis) contribute to the columnar NO system of the FB. Their fibers run first to the PB, and then via the *w*, *x*, *y* and *z* tracts into layer III of the FB and further to the noduli. (b) *tangential neurons*. The single pair neurons of the first tangential system (TL1, see Figure 5Biii) are located in the ventromedian PC. Their neurites ramify in the LBUs and enter the EB via the IT. The second tangential neuron group (TL2) is located in the median PC, the projections are similar to those of the TL1, but they enter the EB more ventrally. The third tangential neuron group (TL3) has arborization fields in the posterior optic tubercle and in the PB. Their somata (about 20) are located posteriorly to the PB. The fourth tangential neuron group (TL4) is composed of six bilateral pairs of neurons which run along the w tract to the LALs. Terminal arborizations are also found in layer II of the FB. The fifth tangential arborization system is localized in posterior commissure I, in layer II of the FB, and in the anterior lip. (c) *Pontine neurons*. These neurons (45 somata in the anterior PI) project through the PB to the FB via the anterior chiasm (according to neuraxis, see Figures 5Biii,iv) and contribute to the columns of layer I and layer IIb in the FB. They also interconnect the columns of these layers.

During development, NADPHd activity first appears at about 70% of embryogenesis in layer IIb of the FB (consistent with the cells of the pontine system being involved), in the EB and in the LAL. At 75%, NADPHd activity appears in columnar neurons in the PI, in the PB, in the columnar y and z tracts, in layer III of the FB, expands via the ITs to the TL2 tangential system and into the posterior groove. (for ventral groove see Kurylas et al., 2005). At 85% of embryogenesis, the staining in the *w, x, y, z* columnar system, in layer III of the FB, in the EB, in the noduli and in the IT intensifies further so that there is a clear resemblance to that of the adult (see Figures 5Bi–iv).

A distinguishing characteristic of the NO system in the developing central complex is that while NADPHd activity in the tangential systems increases in a stepwise manner as more fibers innervate the region, that in the columnar system of the FB appears abruptly and simultaneously in all its elements, suggestive of a temporally coordinated mechanism for establishing this neurochemical architecture (Williams et al., 2005; Herbert et al., 2010).

##### Interim summary

**Figure 7:**
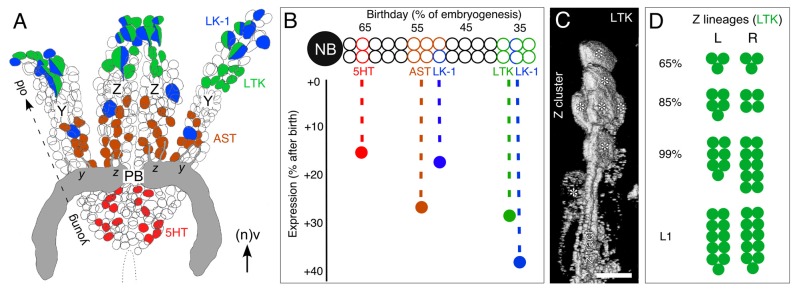
**Topology of neurons expressing various neurochemicals in central complex lineages. (A)** Reconstruction from serial sections following immunolabeling summarizes the location of neurons expressing the neurochemicals 5HT (red), AST (brown), LTK (green) and leukokinin (LK-1, blue) within the bilateral Z and Y lineages at 100% of embryogenesis. Some neurons co-express neurochemicals LK-1 and LTK. Neurons expressing the various neurochemicals are found at stereotypic locations (also representing age) within their lineage: the youngest (latest born) cells are at the base and the oldest at the tip. Neurons direct axons via tract doublets (see Figure 1C) to the PB (gray). Neuroblasts are no longer present at this age (see Boyan and Liu, 2014). Arrow indicates anterior (a) according to the neuraxis (n). **(B)** Schematic illustrates the temporal expression of neurochemicals 5HT (red), AST (brown), LTK (green) and LK-1 (blue) from a representative central complex lineage. Neurons occupy stereotypic locations within the lineage reflecting their birth date. Vertical axis shows the time after birth when neurochemical expression appears. LK-1 cells at the tip of the lineage are born early in embryogenesis but lie immunohistochemically dormant for almost 40% of embryogenesis. 5HT cells at the base of the lineage are born late but express their neurochemical relatively soon thereafter. The data predict there should be a stepwise appearance of neurochemicals within the central complex neuropil. **(C)** 3D image from confocal *z*-stacks following immunolabeling at 80% of embryogenesis shows a cluster of LTK-positive cells (white asterisks) at the tip of the Z lineage along with their projections forming the *z* tract. **(D)** Number of LTK-immunoreactive cells in the left (L) and right (R) Z lineages at various stages of development collated from three preparations in which both Z lineages were completely visualized. LTK-immunoreactive cells make up 2% of the Z lineage at 65% of embryogenesis, 3% at 85%, 5% at 99% and 8% at L1. Scale bar in **(A)** represents 15 μm in **(C)**, 30 μm in **(A)**. Panels **(A,B,D)** modified from Boyan et al. (2010a) with permission.

Several consistent features characterize the developmental expression pattern of neurochemicals in the central complex. First, all the substances tested are expressed in the modules of the embryonic central complex according to a substance-specific temporal pattern, and not synchronously. For some (e.g., 5HT), specific modules (noduli, *w, x, y, z* tracts) only become immunoreactive after hatching, while others (e.g., LTK, AST, NO) appear to have completed their developmental plan during embryogenesis. Second, if we consider individual neuropilar modules, then immunoreactivity appears very early in the LAL and/or the FB, and somewhat later in the PB and *w, x, y, z* tracts. Third, some neuropeptides are expressed earlier (LTK, 50%; AST, 60%) than the NO-synthesizing enzyme diaphorase (70%) or the classical transmitter 5HT (80%). If we focus on just the columnar system, we observe a similar pattern: LTK-related peptides are the first neuroactive substances to appear (70%), followed by AST and diaphorase (NO) at 75–80%, and 5HT only postembryonically. Finally, immunoreactivity does not appear in an all-or-nothing manner during development—there is a clear stepwise increase in signal strength for each neurochemical during development (e.g., LTK, Figure 6A). We can show this (see Figure 7D below) to be due to an increase in the proportion of immunoreactive cells from a given lineage expressing the relevant neurochemical.

### Biochemical Profiling of Central Complex Lineages

Given that the pattern according to which individual neuroactive substances appear during development is critical for the adaptive functioning of the adult nervous system (see Nässel, 2002), knowledge about whether this neurochemical architecture can be related to the lineages of central complex neurons could provide valuable insights into the behavioral role the central complex plays at various critical stages such as hatching, molting, and pupation. Insect lineages of the ventral nerve cord possess an internal temporal topology (see Goodman and Doe, 1994) and this aspect has proven instrumental in integrating neuronal ontogeny and physiology (Goodman et al., 1979, 1980; Taghert and Goodman, 1984; Thompson and Siegler, 1991). It therefore seems relevant to ascertain whether the discrete biochemical layering of the FB neuropil is due to a biochemical zoning within the lineages of neurons, thus reflecting their ages, and suggesting there is a temporal dimension to the biochemical expression pattern associated with central complex neuropils.

Reconstructions have revealed the Y and Z lineages, as representative of central complex lineages, to be bilaterally symmetrical and since they retain their internal organization up to hatching and beyond, they are resolvable to the level of single, identifiable, presumably homologous cells (Boyan et al., 2010a). As a result, the lineages can be shown to possess a temporal topology according to which location within the lineage accords to the birth date of a given cell. This, in turn, allows a lineage to be age-profiled with respect to the expression of various neurochemicals (Figure 6; Boyan et al., 2008a, 2010a), and compare this with the expression patterns of these same substances in the central complex.

The results of such an analysis (Figure 7A) reveal first, that all different neuroactive substances indeed co-localize to the same lineage, implying that the neuroblasts responsible for each lineage are biochemically multipotent. Second, the lineages are almost identically zoned with respect to where neurons expressing these substances are located, suggestive of a common developmental program. LK-1- (a cephalotropin which acts as a circulating hormone modulating visceral muscle contractions and diuresis (Cook et al., 1989; Hayes et al., 1989)) and LTK-expressing cells are clustered apically in each lineage. These cells represent the first-born (oldest) cells of each neuroblast. At the single-cell level, neuroactive substances are also seen to co-localize in some instances. This is not unique as co-expression of neuropeptides has been extensively documented in insect nervous systems (e.g., Taghert and Truman, 1982a,b; Thompson et al., 1995; Duve et al., 2000; Nässel and Winther, 2002), including the central complex (Homberg et al., 1999), although co-localization does not necessarily imply co-release (Marder, 1999).

There is also a prominent second LK-1 expression zone comprising a single large, putatively homologous, cell at an equivalent location midway along each lineage. This younger cell is the evidence to show that the Y and Z neuroblasts have, at the same developmental stage, simultaneously orchestrated a cell division yielding this single LK-1 expressing cell. AST-expressing cells, on the other hand, appear in a continuous zone straddling the central region of each lineage and so are generated by several cell cycles. Serotonergic cells are located only in the basal region of their lineage and so represent the youngest cells of the lineage generated by the last series of divisions of the neuroblast. The clear zoning of expression is the evidence to show that, as in *Drosophila* (Taghert and Goodman, 1984), successive neuroblast divisions generate biochemically distinct cells. The mechanism may involve the neuroblast expressing a transcription factor specific to a given mitotic division or series of divisions thereby providing successive daughter cells with a unique identity (Pearson and Doe, 2003).

All the neuroblasts generating the neurons of the central complex undergo apoptosis between 70% and 75% of embryogenesis (Boyan and Liu, 2014), which means that the central complex of an hemimetabolous insect such as the grasshopper is structurally complete at hatching. Biochemically, however, this is obviously not the case. The brain neurons expressing the various neurochemicals discussed here are all born during embryogenesis, but then remain biochemically dormant for varying periods (Figure 7B). This dormant period is stereotypic for each substance and can last for over 40% of embryogenesis. LTK-expressing cells at the apical tip of the lineage, for example, are born early in embryogenesis (*ca.* 35%), but subsequently lie immunocytochemically dormant for almost 30% of embryogenesis. LK-1-expressing cells also found at the apical tip of the lineage are born over the same time window, but only express the neurochemical about 40% later, i.e., well after the LTK-expressing cells. Serotonergic cells at the base of the lineage are among the last born in the lineage (*ca*. 65% of embryogenesis) but express the neurotransmitter relatively soon thereafter (at 80%).

We reported (in “Developmental Expression Patterns” Section) that immunoreactivity does not appear in an all-or-nothing manner in the FB during development—there is a clear stepwise increase in signal strength for each neurochemical during development. At least in the case of the columnar system, this is most probably due to an increase in the proportion of immunoreactive cells from a given cell cluster of the PI expressing the relevant neurochemical (Figures 7C,D). The cell cluster itself cannot increase in size after 75% because all the proliferative cells responsible for these central complex lineages, including the neuroblasts, ganglion mother cells, and intermediate progenitors, are no longer mitotically active by then (Boyan et al., 2010b; Boyan and Liu, 2014).

### Lineage Topology Translates into Neuroarchitecture

The temporal and biochemical profile of a lineage, coupled with the known times when neurochemicals are expressed in the central complex, allows us to generate a time-line with which to resolve the ontogeny of biochemical expression in the central complex. A concept for how this temporal and biochemical topology might translate into the neurochemical architecture of the central complex can now be formulated.

**Figure 8:**
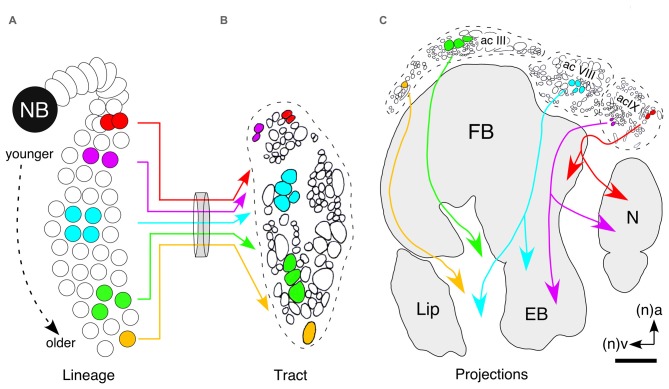
**Hypothesis as to how neuronal topology in a lineage can translate into the topology of columnar projections in the FB. (A)** Neurons occupy stereotypic locations according to birthdate as in this hypothetical lineage of the central complex. Colors are intended to indicate the expression of different, non-specified, neurochemicals. **(B)** Cross-section of the tract shows that axon projections are topologically ordered according to the location of the somata within the lineage. **(C)** Axon position within a tract translates directly into location in commissural fascicles (AC III, VIII, IX) of the central complex. Axons from these fascicles decussate and so generate a columnar neuroarchitecture in the developing FB. In this way the temporal and biochemical topology of the lineage is reflected in the neurochemical architecture of the central complex itself. Arrows indicate anterior (a) and ventral (v) according to the neuraxis (n). Scale bar in **(C)** represents 15 μm in **(B)**. Panel **(B)** modified from Williams et al. (2005); panel **(C)** modified from a personal communication courtesy of Williams.

Neurons occupy stereotypic locations according to birthdate as in the hypothetical lineage of the central complex (Figure 8A). A cross-section of the tract shows that axon projections are topologically ordered according to the location of the somata within the lineage (Figure 8B) and that axon position within a tract translates directly into location in commissural fascicles (AC III, VIII, IX) of the central complex (Figure 8C). Subsets of axons from these fascicles decussate and so generate a columnar neuroarchitecture in the developing FB. In this way, the temporal and biochemical topology of the lineage translates into the neurochemical architecture of the central complex itself.

**Figure 9:**
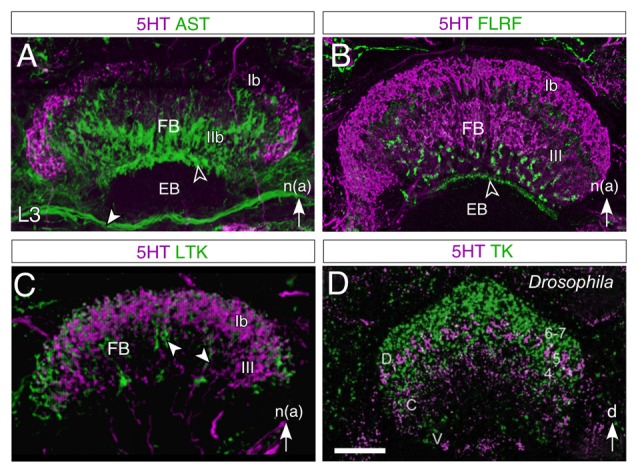
**Layered expression of neurochemicals in the FB of the central complex in the larval (L3) grasshopper (A–C) and adult *Drosophila* (D).** All panels show confocal images following double immunolabeling as follows: **(A)** 5HT/AST, **(B)** 5HT/FLRF, **(C)** 5HT/LTK, **(D)** 5HT/TK. 5HT labeling is magenta throughout. In both species, 5HT is observed not to co-localize to any great degree (lack of white in the image) with the other neurochemicals tested. In the grasshopper, AST labeling **(A)** appears in tangential fibers of region IIb, in an anterior strip (open white arrowhead) of the EB, and in the IT (white arrowhead) between the EB and the MAL (not shown), while 5HT appears as a crescent in region Ib and in the columnar system of the FB; FLRF labeling **(B)** appears in a subregion of III, and in a narrow strip (open white arrowhead) of the EB; LTK labeling **(C)** is present in the Ib and III regions as well as in the columnar system (white arrowheads) but does not colocalize to 5HT. Nomenclature of neuropilar regions is from Vitzthum and Homberg (1998). In *Drosophila* **(D)**, 5HT labeling is present as a thin crescent in layer 5 of the dorsal (D) FB where there may be some minimal co-localization to tachykinin (TK), as well as in central (C) and ventral (V) regions, where there is no co-localization. Abbreviations: (Dip)-Allatostatin I (AST); Phe-Leu-Arg-Phe-NH2 (FLRF); LTK; Tachykinin (TK); 5HT (5HT). White arrow in **(A–C)** points to anterior according to the neuraxis (n). Scale bar in **(D)** represents 35 μm in **(A)**, 65 μm in **(B)**, 95 μm in **(C)**, 25 μm in **(D)**. Panels **(A,B)** modified from Herbert et al. (2010), panel **(C)** modified from Boyan et al. (2010a), panel **(D)** modified from Kahsai and Winther (2011).

**Figure 10:**
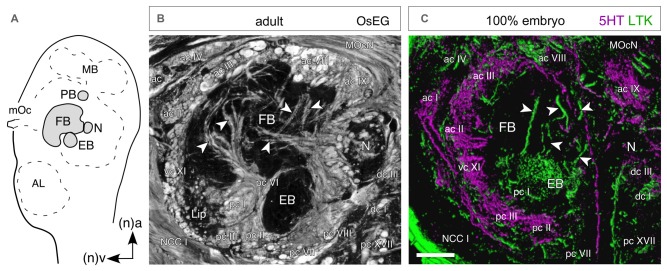
**Temporal topology of neurochemical expression reflects architecture of the central complex. (A)** Schematic (not to scale) shows the location of central complex modules in the brain as seen in sagittal view. Abbreviations: MB, mushroom body; mOc, median ocellus; FB, fan-shaped body; N, nodulus; EB, ellipsoid body; AL, antennal lobe. Arrows point to anterior (a) and ventral (v) according to the neuraxis (n). Axes apply to all panels. **(B)** Histological (sagittal) section of the central complex in the adult brain following osmium ethyl gallate staining reveals axon profiles in anterior (ac), posterior (pc), ventral (vc) and dorsal (dc) commissures circumscribing the FB, EB, N and ventral lip. Other fiber tracts seen are the median ocellar nerve (MocN) and nervus corporis cardiaci I (NCC I). **(C)** Confocal image of a parasagittal section through the central complex at 100% of embryogenesis following 5HT (magenta) and LTK (green) double labeling. There is no co-expression (absence of white) in the commissural and columnar (white arrowheads) neuroarchitecture of the central complex. Since 5HT and LTK are expressed by neurons of different ages (see Figure 7), the co-labeling provides a temporal insight into the neuroarchitecture of the central complex. Scale bar in **(C)** represents 40 μm in **(B)**, 100 μm in **(C)**. Modified from Boyan et al. (2010a).

If our hypothesis that discrete expression zones within the W, X, Y, Z lineages translate into a biochemical neuroarchitecture is correct, then a zoning of neurochemical projection systems might manifest itself as a layered expression pattern in the FB. Indeed, double immunolabeling experiments reveal no co-expression of 5HT on the one hand, and the neuropeptides (Dip)-AST I, FLRFamide, LK-1, PVK/PK and LTK on the other, in arborizations within the same subregions of the FB in either the developing grasshopper (Figures 9A–C; Herbert et al., 2010) or in the case of 5HT and tachykinin, in adult *Drosophila* (Figure 9D; Kahsai and Winther, 2011). Since 5HT and LTK, for example, are expressed by neurons of different ages (see Figure 6), the cellular neuroarchitecture of the FB based on histology (Figure 10B) can now be analyzed via its neurochemical architecture from a temporal perspective (Figure 10C). Late in embryogenesis, ventral commissures, ventral regions of the PB and noduli, are predominantly serotonergic suggesting the axon processes here are from younger cells. The embryonic EB and columnar projections in the FB, by contrast, are almost exclusively LTK-positive, and therefore comprise axons from older cells. This matches the order in which the neuroarchitecture of central complex modules is established (Boyan and Williams, 1997; Williams et al., 2005).

## Conclusions and Outlook

The insect central complex has been shown to orchestrate locomotor and stridulatory behaviors (Strauss, 2002; Kunst et al., 2011), but insect behaviors are not all expressed synchronously during development. They appear sequentially due to induction via steroid hormones (Zitnan et al., 1999), a successive maturation of the neurochemicals themselves (Predel et al., 2003) and the neural circuits responsible (Levine, 1984; Stevenson and Kutsch, 1986; Truman et al., 1996a,b; Wegerhoff et al., 1996). In the holometabolous grasshopper, neural circuits mediating respiratory and skeletal muscle activity must mature during the embryonic phase of development, prior to hatching, and in advance of those regulating, for example, feeding, stridulation or reproduction in the free-living phase (see Chapman, 1982). The temporal differentiation of neurochemicals in the central nervous system reflects this sequence (Goodman et al., 1979; O’Shea and Adams, 1986; Stern et al., 2007).

Clonal analyses of brain neuroarchitecture have been undertaken in larvae and adult *Drosophila* (Ito et al., 1997; Ito and Awasaki, 2008). While these lineages are organized such that cell location accords to birthdate (Lai et al., 2008; Izergina et al., 2009; Riebli et al., 2013) neurochemicals only appear at the adult stage after the central complex neuroarchitecture is established (see Kahsai and Winther, 2011). The mechanisms generating this neuroarchitecture reside at the molecular level with the specification of neurogenesis in the neuroblasts (Doe, 2008) and the guidance cues regulating axogenesis in their progeny (see Dickson, 2002), arguing against a causal role for expressed neuropeptides in the establishment of the central complex. The neuropeptides, monoamines and endogenous messengers expressed in the central complex are also found in other regions of the brain of embryonic insects (Romeuf and Rémy, 1984; Westbrook and Bollenbacher, 1990; Wegerhoff et al., 1996; Bicker, 2001; Seidel and Bicker, 2002). In the case of the grasshopper, however, we can show that these neuroactive substances are expressed in the embryonic central complex at a time when the specific subset of identified neuroblasts responsible for the establishment of its characteristic neuroarchitecture are still present, along with their primary lineages. By linking the neurochemical expression patterns in the developing central complex to the stereotypic location of neurons in these identified lineages, we can relate lineage topology to a developmental plan for establishing the cellular neuroarchitecture of at least one central complex module—the FB.

It is clear that any functional interpretation of the developmental role of specific neurochemicals must be made with caution since neuroactive substances can have different physiological effects in the embryonic nervous system than in the adult, and receptors can be expressed before some of the neuroactive substances themselves are present in the circuit (Roeder, 2002; Rehm et al., 2008). Further, neurochemicals reported for the ventral nerve cord in the adult (e.g., O’Shea and Adams, 1986) or embryonic (e.g., Keshishian and O’Shea, 1985) grasshopper need not play the equivalent role in the development of the central complex in the brain. Since most of the substances examined here are expressed in subsets of tangential and columnar neurons, and some additionally in pontine neurons, our current level of resolution does not allow us to relate a given temporal expression pattern to the functional morphology of individual neuron types. This may eventually prove possible for certain columnar (CPU1, 2) or tangential (TB1a, TB1d) neurons with regionalized morphologies spanning different modules (see Heinze and Homberg, 2008; Pfeiffer and Homberg, 2014; Beetz et al., 2015) if these neurons were to also regionalize their developmental expression of a given neurochemical.

Several other caveats with respect to the data presented in this study must be considered. The first is whether the restricted range of neurochemicals examined in this review is sufficiently representative to draw any major conclusions. Major neurotransmitter systems (glutaminergic, GABergic, dopaminergic and cholinergic), all with clear expression profiles in the adult central complex and established functional roles in behavior (see Homberg, 2002; Kunst et al., 2011; Pfeiffer and Homberg, 2014), are missing. Second, how representative are the W, X, Y, Z lineages for the development of the neurochemical architecture of the central complex *in toto*? We report the expression patterns only up to the first larval stage of development. This is an obvious gap in our knowledge database, and although we see no major neuroarchitectural differences between the central complex of the hemimetabolous grasshopper immediately after hatching and in the adult at the level of resolution available to us (see Boyan et al., 2015), this need not apply at the neurochemical level. Despite the fact that the behavior of the first larval instar has similarities (feeding, locomotion) to that of the adult, there are many differences (reproductive, flight, phase/aggregation; see Chapman, 1982) and these may have their roots at the neurochemical level, even though neural circuits for some behaviors are present at hatching (Stevenson and Kutsch, 1986). For instance, our studies reveal no co-localization of serotonergic and peptidergic cells in embryonic W, X, Y Z lineages (Figure 7), and no cells co-expressing 5HT and neuropeptides in the FB or noduli during embryogenesis (Figures 6, 9, 10). This is clearly not the case in the adult where 5HT and AST are co-localized both in columnar neurons innervating the noduli (Vitzthum et al., 1996; Homberg, 2002) and in clusters of TB neurons projecting into the tract linking the posterior optic tubercle and the PB (Beetz et al., 2015). The lineages of these co-expressing TB neurons have yet to be determined, but as far as the columnar neurons from the W, X, Y, Z lineages are concerned, two possible explanations can be offered: either distinct populations of neurons from the same lineage, but with different biochemical profiles, differentially innervate the FB and noduli, or at least some neurons have changed their biochemical profile during larval development. Axon tracing has yet to clarify the former, but the latter possibility has a precedence in changing peptide co-expression levels during metamorphosis of holometabolous insects (see Honegger et al., 2011) where major structural changes in the central brain (see Wegerhoff and Breidbach, 1992; Renn et al., 1999; Young and Armstrong, 2010a,b; Riebli et al., 2013; Wolff et al., 2015), accompany a neurochemical profile that only reaches maturity at adulthood (Kahsai and Winther, 2011). Nevertheless, our hope is that the temporal sequence of neurochemical expression we uncover in the central complex of the grasshopper also reflects the synaptic maturation of its circuitry, and so may ultimately provide an insight into the way the behaviors it regulates develop.

## Author Contributions

GSB and YL helped with conception, with the text, the figures, and critically reviewed the manuscript.

## Conflict of Interest Statement

The authors declare that the research was conducted in the absence of any commercial or financial relationships that could be construed as a potential conflict of interest.
